# Toward XRF-like chemical sensitivity in the laboratory: first hyperspectral x-ray imaging demonstration using the novel CITIUS detector

**DOI:** 10.1038/s41598-026-48904-6

**Published:** 2026-04-23

**Authors:** V. Di Trapani, P. Thibault, F. Arfelli, Y. Honjo, K. Ozaki, H. Nishino, Y. Joti, T. Hatsui, F. Orsini, R. H. Menk

**Affiliations:** 1 Department of Physics, University of Trieste, Via A. Valerio 2, Trieste, 34127 Italy; 2 Elettra-Sincrotrone Trieste, Strada Statale 14 – km 163.5, Basovizza, 34149 Italy; 3 INFN Trieste, Padriciano, 99, 34149 Trieste, Italy; 4 RIKEN SPring-8 Center, RIKEN, 1-1-1, Koto, Sayo-cho, Sayo-gun, Hyogo, 679-5148 Japan; 5 JASRI, 1-1-1, Koto, Sayo-cho, Sayo-gun, Hyogo, 679-5198 Japan; 6 Department of Computer and Electrical Engineering, Mid Sweden University, Sundsvall, Sweden

**Keywords:** Hyperspectral detector, Spectral imaging, Basis material decomposition, Photon-counting CT, Materials science, Optics and photonics, Physics

## Abstract

X-ray spectral imaging is an advanced technique that enables material-sensitive imaging by exploiting energy-dependent interactions with matter. In this context, X-ray fluorescence (XRF) represents the benchmark technique. Despite its excellent elemental sensitivity, however, XRF is intrinsically inefficient because it relies on the detection of isotropically emitted secondary fluorescence induced by a focused beam on the sample. This requirement makes the technique difficult to implement with compact laboratory sources and, especially for tomographic applications, highly brilliant synchrotron sources are required. In a laboratory environment, spectral imaging and tomography are typically performed using photon-counting detectors. While this technology offers significant advantages, the limited number of energy thresholds and coarse energy resolution can hamper the separation of materials with similar attenuation properties. Hyperspectral detectors, featuring sub-keV energy resolution and virtually unlimited spectral binning, provide a technological solution to enable high-sensitivity, chemical-specific imaging in the laboratory. Here we present the first application of the novel CITIUS hyperspectral detector to X-ray micro-CT and radiography at the OptImaTo (Optimal Imaging and Tomography) laboratory (Trieste, Italy), based on a liquid MetalJet source (Excillum, Sweden) with a galinstan anode. Using multiple characteristic emission lines (Ga, In, Sn) and a ^55^Fe source, a sharp energy resolution in the 0.5–0.8 keV range (full width at half maximum) was found, enabling fine energy binning suitable for advanced quantitative material identification. For the first demonstration, planar and tomographic datasets of two multi-material test samples were analyzed using a newly adapted version of the Minimum-Residual Basis Material Decomposition (MR-BMD) algorithm, optimized for hyperspectral detectors providing tens of energy bins with narrow bandwidths. Results show that the laboratory-based hyperspectral approach combined with MR-BMD enables element-sensitive imaging and, remarkably, separates materials with very similar attenuation, such as water and polypropylene. These results demonstrate accurate material identification and quantification, promisingly approaching XRF-like chemical sensitivity in the laboratory.

## Introduction

X-ray imaging is a non-destructive technique routinely employed to visualize the internal features of samples, which finds application in several fields, including medicine, materials science, geology, and archaeology. By collecting multiple X-ray projections at different angles, computed tomography (CT) is able to provide a three-dimensional map of the attenuation coefficients within the sample. In research and advanced laboratory settings, this is typically implemented as micro-computed tomography ($\mu$-CT), providing micrometric spatial resolution.

In conventional CT, contrast arises from a weighted average of attenuation coefficients over a polychromatic spectrum^1^. As a result, this approach offers a limited ability to differentiate features made by materials with similar attenuation properties. To overcome this limitation and improve material specificity, spectral X-ray imaging techniques have been developed. These methods exploit the fact that, in the energy range of interest for X-ray imaging (5–100 keV), many high-Z materials exhibit sharp discontinuities in their attenuation profiles at characteristic energies (K and L absorption edges). This spectral behavior can be used to enhance contrast and enable material identification.

One of the most established spectral imaging approaches is X-ray fluorescence (XRF), which detects the characteristic secondary emission of elements excited above their absorption edges. XRF is widely regarded as the gold standard for quantitative elemental mapping due to its high specificity and sensitivity^2^. However, despite a few attempts using compact sources^3^, XRF-CT is in practice almost exclusively performed at synchrotron facilities^2^. This limitation arises from the isotropic nature of fluorescence emission and its strong self-absorption in thick samples, which severely reduces collection efficiency and generally restricts laboratory implementations to thin specimens and very high photon fluence rates^4^.

A laboratory-compatible technique for material-sensitive imaging is X-ray spectral imaging with energy-sensitive detectors. This technique aims at material identification and quantification by sampling the energy-dependent attenuation coefficients of materials within a sample at multiple energies. Spectral imaging relies on two main features of X-ray attenuation: (i) the presence of K- (or L-) edges, which appear as a sharp increase in attenuation at specific energies, and (ii) the characteristic energy-dependent attenuation slopes of different materials, resulting from the combination of photoelectric absorption and Compton scattering. By exploiting these distinct attenuation profiles, spectral imaging allows a quantitative chemical mapping of the sample. Although spectral imaging does not reach the same level of specificity as XRF, technological advances in energy-sensitive detectors enable approaching a suitable level of element-resolved imaging and quantification. The main advantage is that spectral imaging can be performed in transmission mode with compact sources, leading to significantly shorter acquisition times and higher suitable photon statistics compared to fluorescence-based methods. This also enables material-sensitive CT of thick/dense samples, where XRF is hardly achievable.

In recent decades, the development of pixelated direct-detection spectral detectors introduced a key advancement in spectral imaging, enabling energy discrimination and sampling into smaller bins simply using a polychromatic source. Spectral detectors are typically categorized into two main types based on their detection architecture: photon-counting detectors (PCDs) and hyperspectral detectors.

Photon-counting detectors process the electrical signal generated by a photon interaction in a semiconductor sensor and compare it to one or more user-defined energy thresholds. These detectors generally feature a limited number of energy thresholds and an energy resolution broader than 2 keV (full width at half maximum at the photopeak)^5–7^, enabling the spectral binning of a polychromatic beam into a few energy intervals, with spectral overlap determined by the energy resolution^8–10^. Owing to their high-rate capability, PCDs are well suited for applications requiring relatively intense X-ray beams, such as biomedical imaging^11,12^ or industrial inspection^13^. However, their limited energy resolution and restricted number of thresholds, resulting in a reduced number of energy samples, hamper the discrimination of materials with similar attenuation profiles or closely spaced absorption edges.

While PCDs represent the most common solution for spectral imaging in clinical and industrial settings, hyperspectral detectors offer a conceptually different approach, providing an alternative solution to overcome the limitations in terms of spectral resolution and sampling of photon-counting architectures, at the cost of reduced counting-rate capability. X-ray hyperspectral detectors are direct-detection, charge-integrating devices that operate at high frame rates, typically in the kiloframes-per-second (kfps) range. In this architecture, the detected signal is proportional to the energy deposited by each photon, and the analysis relies on a low detector occupancy regime, where each charge cluster over neighboring pixels is assumed to originate from a single photon. This enables accurate energy reconstruction and, in principle, super-pixel resolution via center of mass analysis within the cluster^14,15^. With this approach, photons can be sorted into hundreds of user-defined energy bins at the pixel level. These detectors typically achieve sub-keV energy resolution and do not impose a limit on the number of thresholds^15–18^. Their high spectral resolution and fine energy binning enable them to outperform PCDs in tasks such as distinguishing materials with overlapping attenuation profiles or closely spaced K-edges. Moreover, unlike XRF, which relies on isotropic fluorescence and often requires synchrotron-level brilliance, hyperspectral detectors enable both absorption imaging and material identification using compact laboratory X-ray sources^17,19^.

Despite their advantages, hyperspectral detectors face challenges in handling the high fluence rates required for CT, as their integration-based architecture demands high frame rates to resolve single-photon events within pixel clusters^20^. Nevertheless, existing systems have demonstrated feasibility in selected hyperspectral-CT applications with laboratory sources^15,19,21^.

Current hyperspectral detectors present trade-offs in spatial resolution, active area, acquisition speed, and count-rate capability. While high frame rates are critical to manage large photon fluxes, they often come at the expense of reduced spatial resolution or smaller detection areas. However, technological advancements and novel detector designs can mitigate some of these limitations, expanding the applicability of hyperspectral detectors in demanding imaging scenarios.

In this work, a novel large-area hyperspectral detector, named CITIUS, developed by RIKEN and Sony Semiconductor Solutions Corp., is introduced in our experiment. By providing a well-balanced compromise between pixel size, detection area, and counting rate, CITIUS is particularly suited for $\mu$-CT and high-resolution spectral imaging. We report the first demonstration of spectral imaging with CITIUS, performed at the OptImaTo (Optimal Imaging and Tomography) laboratory^22^ (Trieste, Italy) using a microfocus liquid MetalJet D2+ 160 kV X-ray source (Excillum, Sweden). The detector was characterized in terms of energy resolution for both acquisition modes implemented in CITIUS: single-pixel mode (SPM), which enhances spectral resolution by rejecting shared events, and droplet mode (DPM), which preserves detection efficiency through charge-sharing compensation.

To analyze the resulting hyperspectral datasets, we introduce a newly adapted version of the Minimum-Residual Basis Material Decomposition (MR-BMD) algorithm^8^, specifically tailored for datasets from detectors with high energy resolution and a large number of spectral channels. This modified MR-BMD has been designed to accurately decompose materials with similar attenuation properties, even when no K- or L-edge signatures are present within the employed energy range.

As a first imaging demonstration, two representative multi-material test samples were imaged in planar and $\mu$-CT configurations. The results demonstrate that the system can accurately resolve and quantify all selected materials, including water and polypropylene, despite their subtle energy-dependent attenuation differences. The combination of CITIUS and the enhanced MR-BMD algorithm demonstrates the potential to achieve XRF-like chemical sensitivity in the laboratory, where XRF-CT is typically impractical. This approach enables high-specificity material-sensitive $\mu$-CT and opens new opportunities for non-destructive material identification in biomedical imaging, materials science, and cultural heritage.

## Materials and methods

### CITIUS detector

CITIUS is a direct detector made by coupling a 650 μm-thick Si sensor with a CMOS chip implementing integrating-type pixels^23–25^. The chip has an active area of 52.9 mm × 27.9 mm, covered by a matrix of 728 × 384 square pixels with size 72.6 μm. A bias voltage of 500 V is applied to the sensor. The metal block beneath the sensor is maintained at 20 C, which results in the sensor stabilizing at approximately 27 C.

The detection system can operate in two modes: imaging and spectro-imaging. In the imaging mode, the frame rate is set to 17.4 kfps (kilo frames per second), allowing the detection of fluxes up to 30 Mcps (mega counts per second)/pixel (@12 keV). This work focuses on the spectro-imaging mode, which operates at a higher frame rate (26.1 kfps) to allow the detection of single photons in 3 × 3 pixel neighborhoods. In this mode, the detector is configured to acquire trains of 5000 frames with a 36 μs integration time for each acquisition trigger, thereby operating as a hyperspectral detector by enabling energy reconstruction from individual photon events. After data collection, spectral images with energy binning defined by the user are reconstructed with a post-processing analysis of the frames. The post-processing analysis is operated with a dedicated library written in C and parallelized with OpenMP. The library implements two main spectral reconstruction modes: single pixel mode (SPM) and droplet mode (DPM). With the SPM, only non-shared events, where the charge signal is entirely collected by a single pixel, are collected and converted into energy. This algorithm ensures the highest energy resolution at the cost of a loss of detection efficiency arising from the rejection of shared events. The DPM implements a charge summing algorithm to correct for charge sharing, thereby preserving detection efficiency at the cost of increased noise.

**Fig. 1:**
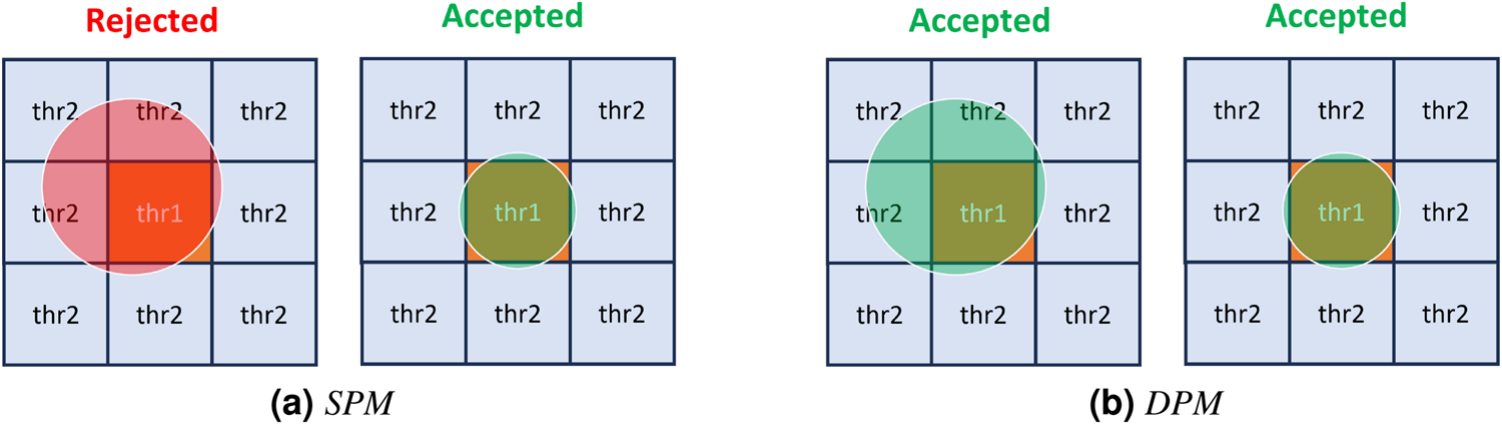
(**a**) single pixel mode (SPM) scheme implementing charge-sharing rejection; (**b**) droplet mode (DPM) implementing charge-sharing compensation for detection efficiency preservation.

Referring to Fig. 1, SPM and DPM algorithms use two thresholds ($thr_1$, and $thr_2$) and operate by applying a sliding 3 × 3 window across the detector image, whereby $thr_1$ is applied to the central pixel and $thr_2$ to the remaining pixels in the window. For both modes, events are considered for processing only if the signal in the central pixel exceeds a threshold $thr_1$, measured in Least Significant Bits (LSB).

In SPM (Fig. 1a), events are rejected if any of the eight neighboring pixels exhibit signals $>thr_2$, indicating charge sharing. Accepted events, where all neighboring pixel signals are below $thr_2$, are converted into energy and stored in the image histogram. In DPM (Fig. 1b), when the central pixel’s signal exceeds $thr_1$, the signals of neighboring pixels with values above $thr_2$ are summed together with the central pixel’s signal. This summed signal is then converted from LSB into energy and stored in the image histogram, ensuring that shared events contribute to the final hyperspectral image, preserving detection efficiency. However, being that the charge-sharing compensation featured by DPM involves the summing of the dark current noise from the summed pixels, a degradation of the energy resolution is expected. In the measurements presented in this work, the thresholds were set to approximately $thr_1 \approx$1 keV for the central pixel and $thr_2 \approx$ 0.5 keV for the neighboring pixels.

### Imaging setup

**Fig. 2:**
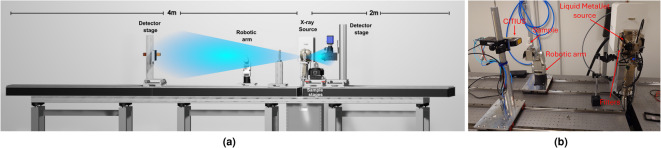
(**a**) Sketch of the OptImaTo laboratory, showing the two branches of the setup: the long branch, used in geometric magnification mode to reach spatial resolutions down to the source spot size (approximately 5 μm); and the short branch, used with high-resolution detectors. (**b**) Photograph of the experimental setup with the CITIUS detector installed on the long branch of the OptImaTo imaging setup.

A prototype of CITIUS has been installed at the OptImaTo laboratory^22^. The laboratory was developed within the framework of the S-BaXIT project, hosted by the University of Trieste and funded by the European Research Council. The imaging setup is built around a high-brilliance liquid MetalJet microfocus X-ray source (Excillum D2+, 160 kV) with a galinstan target alloy^26^. The OptImaTo imaging setup includes two semi-independent imaging branches as shown in Fig. 2a. The short branch (2m long) is dedicated to high-resolution detector-based imaging. The long branch extends up to 4m and is designed for magnification-based cone-beam $\mu$-CT and phase-contrast techniques^22^. CITIUS has been installed in the long branch Fig. 2b, which enables spatial resolutions down to approximately 5 μm, corresponding to the minimum focal spot size of the source. In this setup, samples are mounted on a six-axis robotic arm that serves as both a positioning system and a rotation stage for CT acquisitions.

The energy resolution of CITIUS was evaluated in both SPM and DPM modes, using two complementary sources. A sealed ^55^Fe calibration source with a nominal activity of approximately 3.7 MBq was first used to assess the low-energy response. The source was placed a few centimeters from the detector surface, without additional collimation, as the measurement was intended for spectral calibration only and not for absolute flux quantification. ^55^Fe decays by electron capture to ^55^Mn, emitting characteristic Mn $K_{\alpha }$ (5.9 keV) and $K_{\beta }$ (6.49 keV) X-ray lines. This radionuclide was chosen to provide well-defined low-energy photopeaks below the Cu K-edge, thereby avoiding spectral overlap with the Cu $K_{\alpha }$ fluorescence originating from the detector electronics. To extend the analysis to higher energies, the characteristic lines emitted by the liquid MetalJet source were used directly, without the need for additional targets or external sources. In addition to the continuous bremsstrahlung, the employed X-ray source emits several well-separated peaks originating from its galinstan alloy components: Gallium ($K_\alpha$ = 9.25 keV, $K_\beta$ = 10.26 keV), Indium ($K_\alpha$ = 24.21 keV, $K_\beta$ = 27.28 keV), and Tin ($K_\alpha$ = 25.27 keV, $K_\beta$ = 28.49 keV). These lines span a wide spectral range below 30 keV, matching the sensitive range of the silicon sensor, and providing a convenient approach to evaluate spectral resolution at multiple energies with a single acquisition. For these measurements, the MetalJet source was operated at 70 kV and 40 W, with the detector positioned at a distance of 1 m and a 0.5 mm aluminum filter in the beam. The reference spectra were collected by integrating four acquisition trains, corresponding to a total exposure time of approximately 0.77 s. From the measured polychromatic spectrum, the emission lines were extrapolated by subtracting the bremsstrahlung background. On the isolated peaks, Gaussian fits were performed to extract the full width at half maximum (FWHM) of the photopeaks. This approach allowed accurate estimation of energy resolution without requiring dedicated targets, taking advantage of the source’s characteristic peaks.

**Fig. 3:**
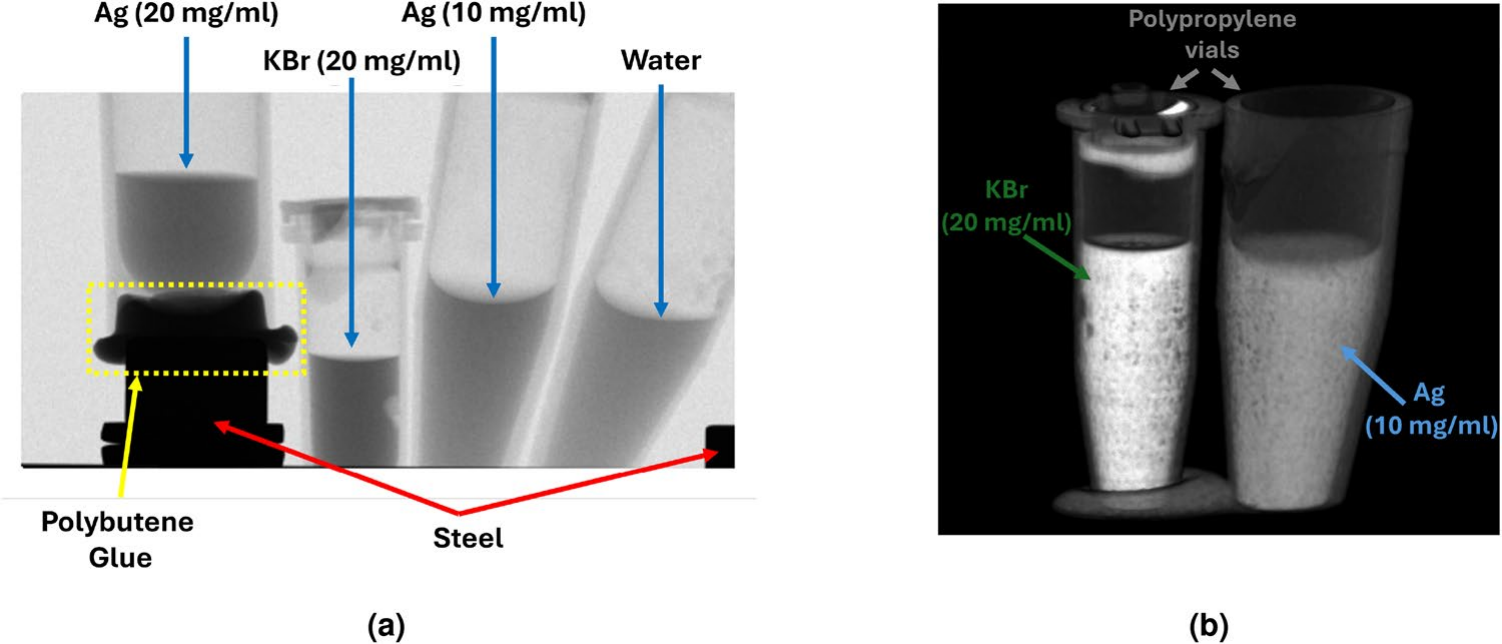
(**a**) Experimental planar radiographic image of the multi-material sample used for spectral imaging, acquired over the full active area of the detector, with all components labeled. (**b**) Experimental 3D volume rendering of the reconstructed $\mu$-CT dataset, showing the polypropylene vials containing Ag/water and KBr/water solutions.

For the first imaging demonstration, two multi-material samples have been assembled. The first, employed for a radiographic image, consisted of a stainless-steel support with two polypropylene vials containing different Ag solutions in water (20 mg/ml, 10 mg/ml), one with a 20 mg/ml KBr water solution, and one with water as reference (see Fig. 3a). The vials were secured to the support using polybutene-based adhesive. For this radiographic image, ten trains of 5000 images were acquired, corresponding to a total exposure time of 1.9s. Spectral images were obtained in DPM, setting energy bins of 0.5 keV in the range 0–35 keV. The second sample, employed for $\mu$-CT imaging demonstration, comprised two polypropylene vials, one filled with 10 mg/ml Ag solution in water and one filled with 20 mg/ml KBr solution in water (Fig. 3b). For both acquisitions, the source was set to 35 kV and 5 W. The source spectrum has been filtered with 250 μm Al + 50 μm Cu. The source-detector and source-sample distances were respectively 75 cm and 67.8 cm, providing a magnification of $M\approx 1.1$ and an effective pixel size of 65.6 μm. Tomographic data were acquired over 360 angular positions uniformly distributed over 360° , with each projection consisting of ten trains of 5000 frames, resulting in an exposure time of approximately 1.9 s per projection and a total scan time of about 689 s.

### Spectral decomposition framework

The spectral images were processed using the MR-BMD approach presented in^8^. The MR-BMD algorithm belongs to the family of basis material decomposition methods, where acquiring the same image at different energies allows solving a system of *m* equations with *n* unknowns, where $n \le m$^27^. The original MR-BMD implementation operates in the projection domain, with the system expressed as follows:

1 $$\begin{aligned} \begin{bmatrix} -\log \left[ \frac{I}{I_0}(E_1)\right] \\ -\log \left[ \frac{I}{I_0}(E_2)\right] \\ \vdots \\ -\log \left[ \frac{I}{I_0}(E_N)\right] \end{bmatrix} = \begin{bmatrix} \left[ \frac{\mu }{\rho }(E_1)\right] _1 *\delta (E_1) & \cdots & \left[ \frac{\mu }{\rho }(E_1)\right] _M *\delta (E_1) \\ \left[ \frac{\mu }{\rho }(E_2)\right] _1 *\delta (E_2) & \cdots & \left[ \frac{\mu }{\rho }(E_2)\right] _M *\delta (E_2) \\ \vdots & \ddots & \vdots \\ \left[ \frac{\mu }{\rho }(E_N)\right] _1 *\delta (E_N) & \cdots & \left[ \frac{\mu }{\rho }(E_N)\right] _M *\delta (E_N) \end{bmatrix} \cdot \begin{bmatrix} (\rho \xi )_1 \\ (\rho \xi )_2 \\ \vdots \\ (\rho \xi )_M \end{bmatrix} \end{aligned}$$

In Eq. 1, $I(E_i)$ and $I_0(E_i)$ denote the measured and reference intensities at the energy bin $E_i$. The term $\left( \mu /\rho \right) _m$ represents the mass attenuation coefficient of the $m^{th}$ basis material, while $*\delta (E_i)$ accounts for the system’s spectral response at energy $E_i$, here included as a Gaussian broadening that models the finite detector energy resolution. The unknown density–thickness product $\rho \xi$ is then obtained by solving the system using a standard least-squares fitting approach, yielding *M* quantitative images: one for each basis material.

The distinctive feature of the MR-BMD algorithm lies in its two-step design. First, the least-squares residuals are computed for all possible combinations of basis materials. Then, for each pixel, the combination that minimizes the residual is selected. As demonstrated in^8^, this pixel-wise minimization of the number of basis materials imposes sparsity on the decomposition basis and improves the stability of the inversion. Specifically, reducing the number of basis functions lowers the condition number of the inverse problem, leading to more robust and less noisy solutions. The MR-BMD algorithm also minimizes cross-talk between the decomposed material channels, especially under low-photon conditions or similar attenuation properties of the basis materials^8^.

In this work, the MR-BMD algorithm was adapted to hyperspectral detectors, which provide a large number of narrow energy bins with sub-keV spectral resolution. This configuration allows for the assumption of quasi-monochromaticity for each energy bin. However, fine energy sampling impacts the photon statistics, often leading to increased noise or even empty bins. To address this, we solve the nonlinear system described by the Lambert–Beer law:

2 $$\begin{aligned} I(E_i) = I_0(E_i) \cdot \exp \left( -\sum _k \left[ \left( \frac{\mu _k(E_i)}{\rho _k} *\delta (E_i) \right) \cdot (\rho \xi )_k \right] \right) \end{aligned}$$

where $\mu _k(E_i)/\rho _k$ are the tabulated mass attenuation coefficients from the NIST database XCOM^28^, and $(\rho \xi )_k$ are the unknown products of density and thickness for the $k^{th}$ material. These quantities, representing the total amount of each material along the X-ray path, are estimated by solving a constrained nonlinear least-squares problem using the SciPy library^29^, with non-negativity constraints and upper bounds set according to the estimated/measured maximum sample thickness.

It is important to note that, also in this modified implementation, the two-step basis selection process of MR-BMD is retained. Therefore, for each pixel, all candidate basis combinations are probed, and the one yielding the smallest residual is used for the final decomposition in the pixel itself. This ensures robustness even with low statistics and improves the separation of materials with similar spectral signatures, such as K-edge jumps close in energy, or similar energy-dependent slopes.

In the CT case, once all projections are processed, the $\xi$ dependence is effectively removed through tomographic reconstruction, resulting in separate volumetric maps of the material densities $\rho _k$ for each basis component.

A distinctive feature of the present MR‑BMD implementation is that the decomposition is applied directly on the projection data, before CT reconstruction. This diverges from the typical approach adopted in most spectral CT studies with hyperspectral detectors, where reconstruction is first performed for each energy bin and decomposition is subsequently applied. Spectral analysis methods commonly used on reconstructed images include K-edge subtraction and absorption step-size fitting^17,19^, which focus on identifying K-edge jumps of the attenuation coefficients associated with high-Z elements.

Spatio-spectral regularization techniques have recently shown promising results in improving robustness when working on reconstructed volumes, particularly under noisy or under-sampled conditions^30^. However, these methods involve a fine-tuning of the regularization parameters and extended processing times. In our approach, material decomposition is performed directly on the raw projections instead, thereby avoiding reconstruction-related artifacts under low-photon conditions.

Importantly, the MR-BMD approach is not limited to K-edge identification, but exploits the full energy-dependent attenuation profile, including its slope, to distinguish materials even when no K-edges are present within the employed energy range. As demonstrated in the results section, this allows for the identification of spectrally similar components, such as water and common polymers, which are typically difficult to separate with conventional edge-based methods.

## Results

**Fig. 4:**
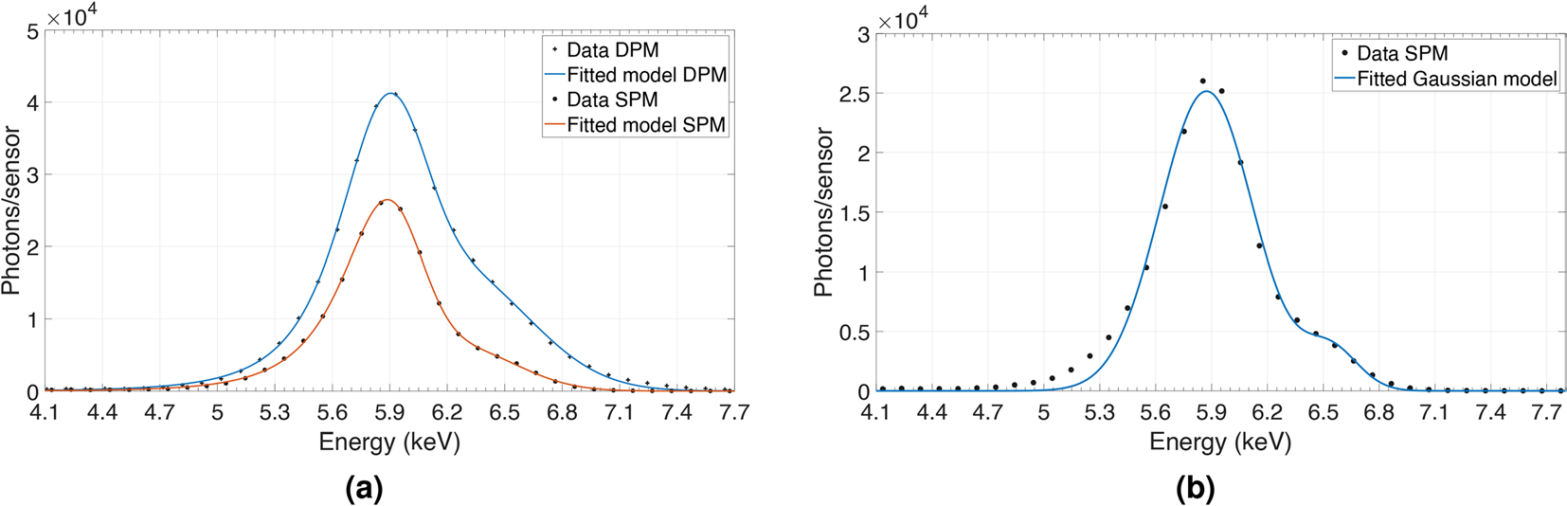
(**a**) Spectra of the ^55^Fe source acquired in both SPM and DPM modes, fitted using the modified Hypermet function. The fit provides a good agreement with the experimental data, capturing the low-energy tail due to incomplete charge collection. (**b**) Example of Gaussian fitting on the SPM spectrum, showing the poor match and the inability of the model to reproduce the asymmetric peak shape.

The spectral response of the detector was first characterized using a ^55^Fe source. The acquired spectra in both SPM and DPM modes are shown in Fig. 4a. As shown in the example in Fig. 4b, for these energies, the standard Gaussian model failed to accurately reproduce the experimental peak shapes, due to the left tail caused by incomplete charge collection, primarily influenced by the thresholds applied in both SPM and DPM modes. To account for the low-energy tail featured in the detected spectra, the two Mn peaks were fitted using the modified Hypermet function^31^. This function takes into account the incomplete charge collection, providing a significantly better match to the data, as illustrated in Fig. 4a. Referring to Mn spectra, for the estimation of the energy resolution, only the $K_\alpha$ peak was used, since the overlap between the weaker $K_\beta$ peak with the $K_\alpha$ peak prevents a reliable FWHM evaluation. The measured energy resolution for both modes is reported in Table 1.

**Fig. 5:**
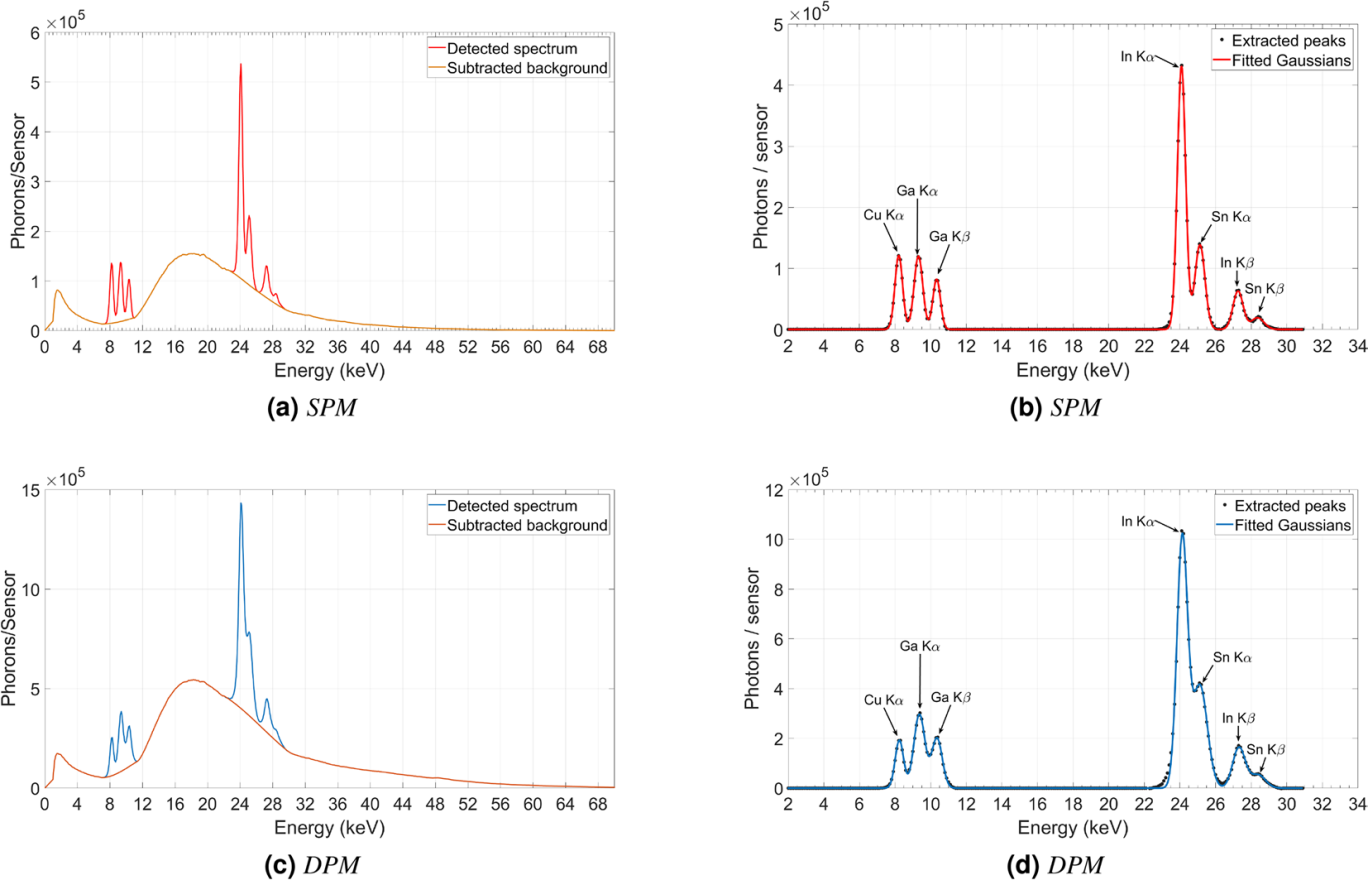
Spectral analysis of the characteristic emissions from the liquid MetalJet source for both acquisition modes. (**a**) Raw spectrum acquired in SPM mode showing the bremsstrahlung background and the characteristic peaks from Ga, In, Sn, and Cu. (**b**) Isolated peaks after background removal and corresponding multi-Gaussian fit (7 peaks). In (**c**) and (**d**), the corresponding analysis is presented for the DPM acquisition mode.

The energy resolution at higher energies was evaluated using the characteristic emissions of the liquid MetalJet source, which, in addition to the bremsstrahlung background, provides sharp emission lines from gallium, indium, and tin, spanning a broad energy range (9–28.5 keV). In Fig. 5a, it is shown how the characteristic emission lines have been extrapolated, removing the Bremsstrahlung background using the modified Akima interpolation method^32^, implemented in MATLAB (The MathWorks, Natick, USA).

Among the energy peaks detected by the imaging system in Fig. 5b, it can be noted that an additional emission line is observed at 8 keV, corresponding to the $K_\alpha$ fluorescence of copper. This emission originates from the chip-to-sensor interconnects in the detector architecture and is consistently present in both SPM and DPM spectra. In this case, for all the detected energies, the effects of the incomplete charge collection appear to be mitigated by the higher energy, and no relevant deviations from a Gaussian response have been noted. The spectra and fits for both SPM and DPM modes are shown in Fig. 5, and the results for all the analyzed energy peaks are summarized in Table 1.

**Table 1:** Measured energy resolution (FWHM of full-energy peaks) for SPM and DPM modes on the characteristic peaks of the ^55^Fe source and the liquid MetalJet source.

Source	E (keV)	FWHM (keV) SPM	FWHM (keV) DPM	Efficiency SPM/DPM (%)
Fe^55^ (K_*α*_ Mn)	5.9	0.54 ± 0.01	0.60 ± 0.05	52 ± 3
K_*α*_ Cu	8.0	0.51 ± 0.01	0.55 ± 0.03	58 ± 7
K_*α*_ Ga	9.3	0.57 ± 0.01	0.73 ± 0.03	32 ± 3
K_*β*_ Ga	10.3	0.50 ± 0.01	0.70 ± 0.04	30 ± 4
K_*α*_ In	24.2	0.54 ± 0.01	0.70 ± 0.03	34 ± 1
K_*α*_ Sn	25.3	0.66 ± 0.01	0.82 ± 0.04	32 ± 2
K_*β*_ In	27.3	0.67 ± 0.02	0.83 ± 0.06	31 ± 6

Uncertainties on FWHM values correspond to the 95% confidence interval obtained from the Gaussian (or Hypermet) fit. The relative detection efficiency of SPM was estimated as the ratio of the integrated area of each fitted peak with respect to DPM; uncertainties were propagated from the fit parameters using standard error propagation.

From the results in Table 1, as expected, SPM mode outperforms the DPM in terms of energy resolution. The energy degradation in DPM is due to the summation of noise across multiple pixels. The relative detection efficiency of SPM with respect to DPM was estimated by integrating the area under each fitted Gaussian peak and computing the SPM/DPM ratio. These values are reported in the last column of Table 1. The results show that on average, SPM collects approximately 30% of the events compared to DPM. From these results, it follows that DPM is better suited for imaging applications, where improved statistics naturally yield improved image quality featuring higher signal-to-noise ratios (SNR). In contrast, SPM remains the best option when high spectral resolution is required for both imaging and spectroscopic measurements.

**Fig. 6:**
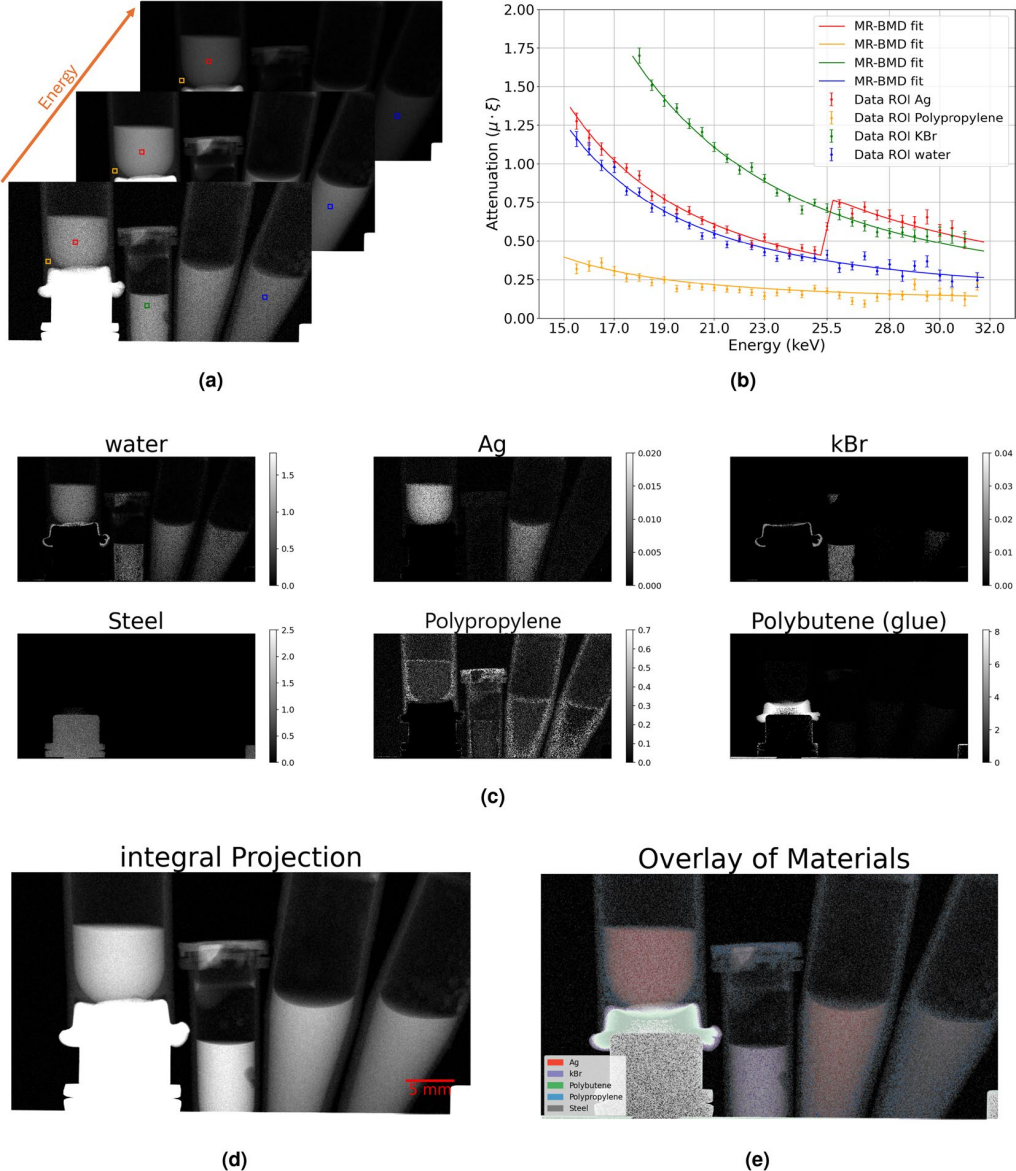
(**a**) Sketch illustrating a hyperspectral dataset as a stack of projections at increasing energies, with decreasing attenuation and selected ROIs for four materials: polypropylene (orange), Ag 20 mg/ml (red), KBr 20 mg/ml (green), water (blue). (**b**) Pixel-averaged $\mu (E)\cdot \xi$ and MR-BMD fits for each ROI, showing clear energy-dependent signals and accurate matches; the error bars represent the standard deviation of the mean over the 10 × 10 pixel ROIs. (**c**) Individual basis material maps from MR-BMD decomposition. (**d**) Conventional projection obtained by integrating all detected photons from the hyperspectral dataset. (**e**) Composite image with color overlay of the decomposed basis materials.

Figure 6 summarizes the complete MR-BMD processing workflow, from hyperspectral input to quantitative material decomposition of the first multi-material test sample shown in Fig. 3a.

In Fig. 6, panel (a) displays representative projections at increasing photon energies, extracted from the full hyperspectral dataset composed of multiple energy channels. These images illustrate the energy-dependent attenuation behavior of the sample. To demonstrate the spectral characteristics of the dataset, four 10 × 10 pixel regions of interest (ROIs) were defined on a few selected materials, i.e., polypropylene (orange), Ag 20 mg/ml (red), KBr 20 mg/ml (green), water (blue). The corresponding attenuation curves and the resulting MR-BMD fits are shown in panel (b). It is important to note that these results have been averaged over each ROI to reduce statistical fluctuations and clearly highlight spectral trends. Although this averaging is used here for representation purposes in (b), all decompositions were performed on a pixel-by-pixel basis, being affected by increased statistical noise. Nevertheless, panel (c) demonstrates that the MR-BMD algorithm is effective in identifying all materials in the sample, including those with similar attenuation profiles such as water and polypropylene. For reference, panel (d) shows the conventional projection obtained by integrating all detected photons. Panel (e) presents a color overlay of the decomposed basis materials, highlighting the achieved material identification.

To further validate the performance of the approach under more complex conditions, the same MR-BMD algorithm was applied to tomographic data acquired from the second multi-material phantom in Fig. 3b. In this case, the challenge lies in the volumetric nature of the dataset and the overlap between materials along the beam path occurring in the tomographic acquisition.

**Fig. 7:**
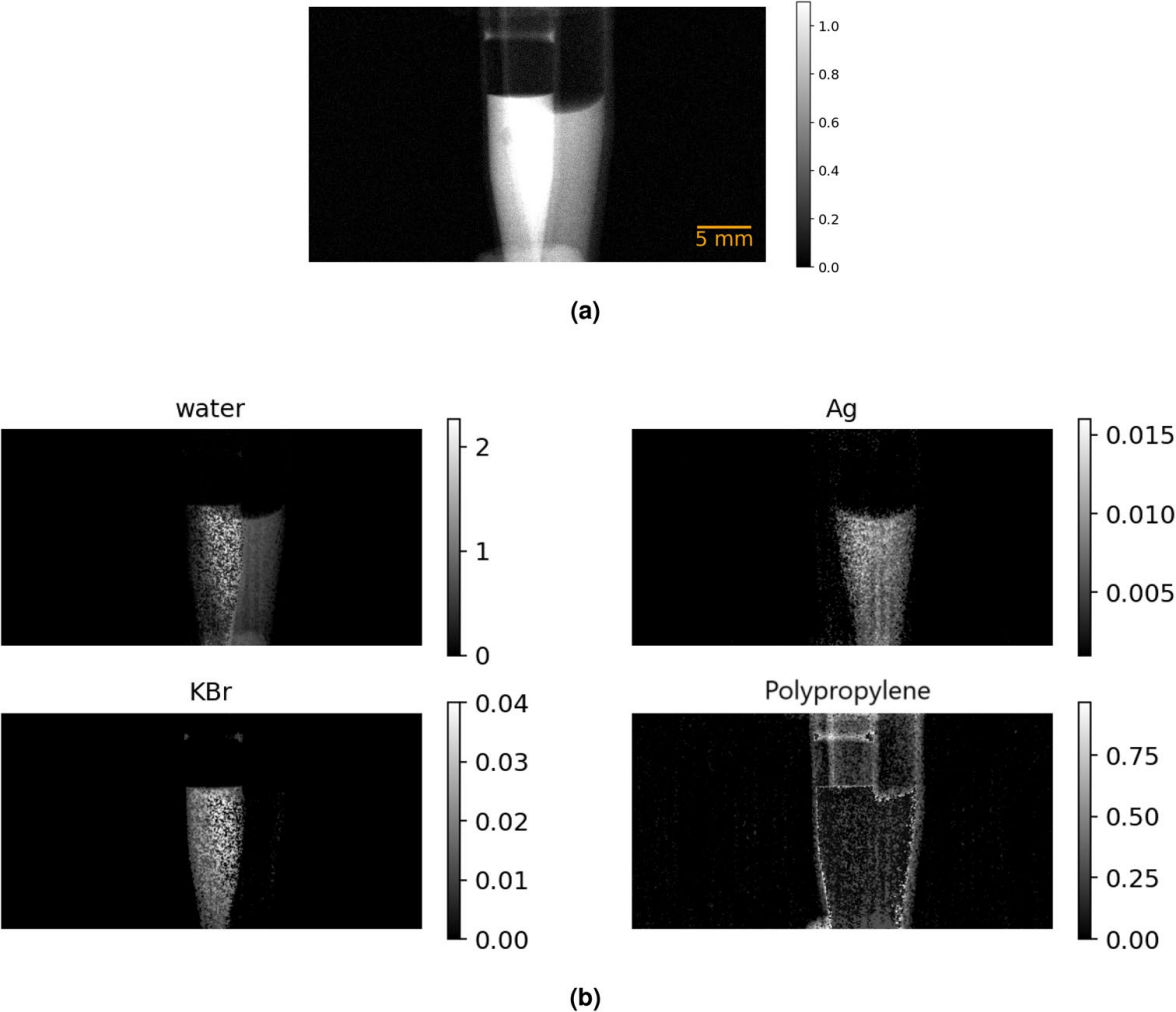
(**a**) Representative projection image from the $\mu$-CT acquisition, showing a partial overlap between the two vials filled with Ag and KBr solutions. (**b**) Spectral decomposition results, using the MR-BMD algorithm, successfully separating the contributions of Ag, KBr, water, and polypropylene, even in regions of all four materials overlap.

For each projection of the $\mu$-CT dataset, the MR-BMD algorithm was applied to perform material decomposition. Figure 7 shows a representative example. In panel (a), the raw projection shows a region where Ag and KBr solutions partially overlap, creating a challenging case for spectral unmixing. Panel (b) displays the MR-BMD output, which successfully resolves the contribution of each material, even in areas where all three basis components overlap. This performance is enabled by the combination of the MR-BMD algorithm and the high-resolution hyperspectral dataset provided by CITIUS, which allows robust per-pixel selection of the optimal basis subset even under strong spectral mixing and low SNR.

From Fig. 7b, it can also be observed that in the central region of the vials, the polypropylene signal is noisy and not fully recovered, as the liquid contribution dominates the attenuation. This challenging case, where one component prevails along the beam path, is a known issue and will be further addressed in future developments of the MR-BMD algorithm. Importantly, at the edges of the vials, where the liquid layer is thinner, the polypropylene contribution is consistently recovered.

**Fig. 8:**
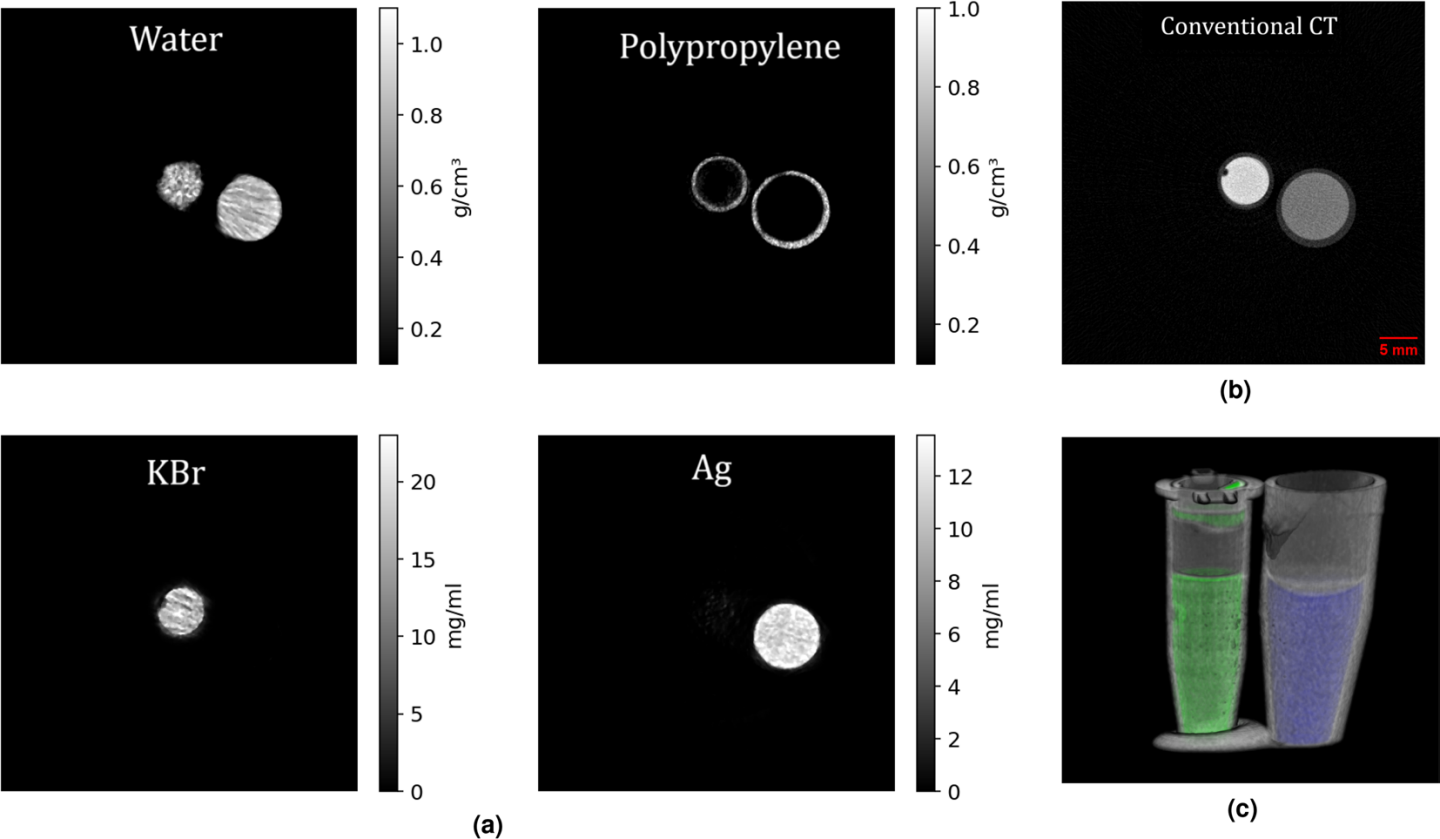
(**a**) Quantitative $\mu$-CT material decomposition of four components: water, polypropylene, KBr, and Ag. (**b**) Conventional $\mu$-CT image obtained by integrating all detected photons from the hyperspectral acquisition. (**c**) 3D rendering of the spectral decomposition: KBr solution in water (green), Ag solution in water (blue), and polypropylene tube walls (gray).

The decomposed projections were reconstructed using the Maximum Likelihood Expectation Maximization (MLEM) algorithm implemented in the TIGRE toolbox^33,34^, which is well suited for low-statistics datasets and demonstrated robust performance in this context. The reconstruction of the $\mu$-CT sample is shown in Fig. 8. In this case, each voxel represents the density of each material, providing a quantitative demonstration of the decomposition.

As shown in Fig. 8, all four materials are correctly identified and localized in the reconstruction, confirming the reliability of the decomposition. Notably, the reduced polypropylene signal observed in the central region of the vials in the projections does not affect the final CT reconstruction, as the vial walls are accurately recovered and clearly identified.

Quantitative results, reported in Table 2, represent the mean densities measured over five 10 × 10 pixel ROIs selected in consecutive slices. The uncertainties indicate the standard deviation across the ROI means and reflect the detection limit under the given acquisition conditions. The expected values correspond to reference densities from NIST for water and polypropylene, and to nominal concentrations prepared in the laboratory using calibrated precision scales for Ag and KBr.

**Table 2:** Quantitative estimation of material densities and concentrations from the tomographic dataset, as obtained by MR-BMD decomposition followed by MLEM reconstruction. Values represent the mean over five ROIs (10 × 10 pixels) selected in consecutive slices, while uncertainties denote the standard deviation across these ROI means. The measured values show good agreement with the expected densities and concentrations for all materials.

Material	Measured density/concentration	Expected
Water	$1.0 \pm 0.2$ g/cm^3^	1 g/cm^3^
Polypropylene	$0.9 \pm 0.1$ g/cm^3^	0.9 g/cm^3^
Ag	$11 \pm 2$ mg/ml	10 mg/ml
KBr	$21 \pm 2$ mg/ml	20 mg/ml

From the results in Table 2, it can be noted that although the estimated values are in good agreement with the expected ones, the associated uncertainties are relatively large, with relative errors typically in the range of 10–20%. This is not an intrinsic limitation of either the hardware or the material decomposition algorithm, but rather a consequence of the low-photon statistics deliberately used in this first demonstration. As a first limitation, the source was operated at 5 W, well below the linearity limit of the system, to ensure conservative acquisition conditions, while subsequent tests showed that the same imaging protocol can be safely performed up to 30 W, potentially increasing the photon statistics by a factor of six. Another major limitation was the data transfer time associated with spectral acquisition. The total scan time was of approximately 689 s (1.9 s exposure per projection), but the overall acquisition required nearly 6 h. This long acquisition time was primarily due to the delay in transferring data from the detector to the dedicated server. This was a known constraint of the current CITIUS prototype hardware. These issues are expected to be overcome in the next version of CITIUS, where spectral reconstruction of raw frames will be performed directly on the FPGA^35^. This will significantly reduce the data volume to be transferred and is expected to drastically shorten acquisition times, enabling faster and more efficient scans under higher fluence rate conditions.

## Conclusions

This work presented the first demonstration of hyperspectral X-ray imaging and chemical decomposition using the novel CITIUS detector in spectro-imaging mode, demonstrating its suitability for material-sensitive $\mu$-CT and radiography. By combining CITIUS with the MR-BMD algorithm, we achieved accurate material decomposition, resolving not only elements with K-edge features but also challenging low-Z materials with similar attenuation slopes, such as water and plastics. This capability significantly expands the range of chemically distinguishable materials in laboratory-based hyperspectral imaging, overcoming a key limitation of conventional approaches typically restricted to K-edge fitting^17,19,30^. Importantly, the MR-BMD algorithm is applied directly to the projection data, with tomographic reconstruction performed afterward on the decomposed channels. This strategy avoids reconstructing narrow energy bins individually, which would otherwise fragment the photon statistics over many bins, leading to noisy CT volumes and artifacts, including those from photon starvation, that can compromise the accuracy of the spectral decomposition

The main limitation in this study was the long dead time between acquisitions, caused by the data transfer bottleneck in the prototype hardware. To address this issue, the next-generation CITIUS will integrate FPGA-based spectral processing^35^, which is expected to drastically reduce data transfer volumes, thereby enabling faster, continuous hyperspectral CT acquisitions with improved photon statistics.

More broadly, this work contributes to the ongoing development of laboratory-based X-ray hyperspectral $\mu$-CT setups, complementing previously proposed solutions that target sharp energy resolution in the high-energy range using thick high-Z sensors (such as CdTe) and large pixel sizes^18,36^, or fine-pitch detectors designed to enhance super-pixel spatial resolution, though typically limited in energy resolution and active area^15^. The CITIUS prototype employed in this work offers a balanced trade-off between pixel pitch, energy resolution, and active area. However, it should be noted that the 650$\mu$m silicon sensor inherently restricts its operational range to low- and medium-energy X-rays (e.g., below approximately 30 keV), where the detection efficiency remains adequate, while it decreases rapidly at higher energies.

The results presented here represent a first step toward practical laboratory-based hyperspectral imaging with CITIUS, achieving high specificity in material identification. Future developments will focus on increasing acquisition speed, increasing photon statistics, improving MR-BMD performance, and validating the system on application-relevant samples.

## Data Availability

The datasets used and/or analyzed during the current study available from the corresponding author on reasonable request.
